# Lived experiences of women suffering from Tuberculosis in Kashmir: an interpretative phenomenological study

**DOI:** 10.3389/fgwh.2025.1464091

**Published:** 2025-03-13

**Authors:** Rubeena Akhter, Wakar Amin Zargar, Fayaz Ahmad Paul

**Affiliations:** ^1^ Centre for Women Studies and Research, University of Kashmir, Srinagar, India; ^2^ Department of Social Work, University of Kashmir, Srinagar, India; ^3^ Department of Psychiatric Social Work, LGB Regional Institute of Mental Health, Tezpur, India

**Keywords:** Tuberculosis, women, conceiving, motherhood, Kashmir, illness

## Abstract

**Objectives:**

To investigate women's experiences with tuberculosis and the challenges they face during their illness. It also explored the impact of infertility on their social relationships.

**Method:**

The study used Interpretative Phenomenological Analysis to understand the experiences of 21 women affected by tuberculosis. These women shared their tuberculosis-related infertility struggles and the repercussions they faced during their illness.

**Results:**

The results are discussed in terms of two main themes: 1. Tuberculosis as an enduring experience with an emotional response to physical suffering; and 2. Tuberculosis and the concern of conceiving as a case beyond Microbiology.

**Conclusion:**

The study findings provide better insight into the sufferings and miseries of infertility due to tuberculosis, as well as the impact of illness on overall well-being, emphasizing the need for targeted intervention.

## Introduction

Becoming a mother is one of humankind's simplest and most complex identity-development tasks. Motherhood encompasses the stages of expecting, childbearing, parenting, and becoming a mother, all of which can provide a woman with a sense of realization and accomplishment (1). Although motherhood has achieved a significant place in the social milieu, it is one of the most explored, conjectured, and discussed issues in feminist theory (2). The feminist movement has enriched, confronted, and transformed perceptions of motherhood since its rise (3). Moreover, early second-wave feminists argued that eradicating motherhood in its current form would completely transform social relations and social production (2, 4). While broader gender dynamics shape the experiences of individuals with tuberculosis, adopting a feminist perspective allows for a deeper understanding of the unique societal pressures faced by women. In the socio-cultural context of Kashmir, women's identities are often closely tied to their reproductive roles and caregiving responsibilities. A feminist lens highlights how these societal expectations exacerbate the stigma, discrimination, and psychosocial burden faced by women suffering from tuberculosis, particularly those dealing with infertility. This perspective is essential for understanding the gender-specific challenges that extend beyond the biological effects of the disease. Ruddick (2009) held the view that women's journey and understanding of motherhood shape their thoughts and behaviour in observing the world, a concept she refers to as “maternal thinking” (5).

Ruddick (2009) identified that maternal thinking differs from war and violence and rests on the major belief that the mother is the key source of attention, support, protection, and shelter for children. Motherhood holds a significant and protective position in a woman's life, primarily due to its social significance; however, it also serves as a full-time profession that separates a woman from herself. Perceptions of motherhood have been the distinctive social need for extended care and nursing of infants has taken for granted perceptions of motherhood (6, 7). Motherhood and mothering vary across different cultures, civilizations, societies, humanities, and over history (6).

For many women, the phase of motherhood is associated with grief, misery, sorrows, burdens, suffering, ill health, and occasionally even death (8, 9). It is a very challenging phase for the reason that every minute of every day, a woman dies It is a particularly challenging phase due to the fact that complications during the phase of motherhood cause a woman's While motherhood is a natural process that brings joy to individuals, women, and families, it is not without risk (10, 11). Contracting deadly illnesses during this phase can significantly impact women's health, and for many, becoming a mother while grappling with life-threatening illnesses can pose significant challenges (8). Motherhood in the context of a long-term illness such as tuberculosis is a seldom-discussed topic in both research and medical practices.

The tuberculosis epidemic and the population it affects undergo continuous changes that mutually affect each other (12). People not only recognize tuberculosis as an individual health problem, but also as a psychosocial suffering that can lead to the neglect of basic rights for affected patients (13–15). Women have experienced particularly severe outcomes, including the trauma of divorce, desertion, isolation, ostracism, anxiety, depression, fear, abandonment, the transmission of illness to infants, and separation from children, all of which negatively impact child survival and family welfare (7, 16). Fears of contracting tuberculosis frequently result in isolating situations, such as compelling a tuberculosis patient to distance herself from her spouse, thereby depriving them of their emotional and sexual rights, and to separate from their children, thereby compromising the essence of motherhood (16–19).

## Materials and methods

The study aimed to examine and inquire into the experiences of women with tuberculosis, as well as the gloominess and despair they faced because they were unable to conceive due to illness. Consequently, this study adopted a qualitative approach, aiming to gain a deeper understanding of human sufferings, social relationships, and the intricacies of the disease through a thorough investigation of patients' actual experiences of tuberculosis infection, the inability to conceive, and the subsequent life challenges. Therefore, we employed interpretative phenomenological analysis (IPA) to address the research question, capturing the factual experiences of patients diagnosed, affected, and currently living with tuberculosis. Our main goal was to learn about the in-depth experiences of women with tuberculosis, how they handled their knowledge, and what challenges they faced. Thus, this phenomenological method of inquiry entails a thorough understanding of study participants' perspectives on illness and provides fact-finders and researchers with the best opportunity to recognize and figure out the deepest, most classified, and most constrained thoughts of the lived experiences of research participants.

## Participant recruitment

As far as the present study is concerned, the study participants were married women affected by tuberculosis. The present study's participants were unable to conceive due to genital tuberculosis, a condition they discovered after undergoing specific diagnostic tests such as endometrial biopsy, polymerase chain reaction (PCR) testing, laparoscopy, and hysterosalpingography, which are standard procedures to confirm genital tuberculosis and its association with infertility. Participants with genital tuberculosis (TB) were identified through referrals from hospitals and specialized TB clinics across the Kashmir division. The study employed purposive sampling to select participants, focusing on married women aged 15–49 who were in their reproductive phase and had been clinically diagnosed with genital TB. This age group was chosen because, according to medical terminology and infertility statistics, this phase falls under the reproductive phase of women (20). Participants were recruited from both rural and urban areas to capture a comprehensive understanding of their experiences within different socio-cultural contexts. Furthermore, the study included 21 married people from rural and urban areas of the Kashmir division. Among the participants, 52.4% were from rural areas, and 47.6% were from urban areas, ensuring a balanced representation across diverse backgrounds. It is important to note that there is no precise answer to the question of sample size in interpretative phenomenological analysis. However, Smith et al. (2009) and Bhat (2014) have also suggested the use of less concentrated samples for idiographic commitment (21, 22). Therefore, after conducting and investigating the interview processes, the theoretical saturation of the data reached 21. Before collecting data and conducting interviews with study participants, we thoroughly discussed the study's purpose and sent informed consent to the participants to uphold the study's ethical standards. However, those participants who were in the age group of 15–49 were considered for the present study, as this age group falls under the reproductive phase of a woman.

## Data collection

The present study collected data through semi-structured interviews. Interviews were conducted at the participants' residences to ensure convenience, comfort, and a familiar environment for the participants, many of whom were dealing with physical discomfort due to tuberculosis. Conducting interviews in their homes reduced the burden of travel and potential stigmatization associated with attending interviews at public facilities. To address concerns regarding privacy and potential influence from family members, efforts were made to ensure that interviews took place in private settings within the home. Participants were also given the option to reschedule if privacy could not be ensured during the initial session. A semi-structured interview format was chosen to allow for flexibility and in-depth exploration of participants' personal experiences, emotions, and perceptions. This approach provided space for participants to share sensitive information at their own pace, enabling a deeper understanding of the psychological, social, and emotional impacts of tuberculosis. We examined the study participants to understand their perceptions, thoughts, and experiences of tuberculosis, their inability to conceive due to genital tuberculosis, and the impact of the disease on their overall well-being. We segregated the research questions in this study based on how tuberculosis affects conception. This includes the study participant's experience of not being able to conceive because of the disease, as well as how the disease has affected her desire for motherhood. The interview process started with an opening question: 'State us about your experiences as a tuberculosis patient and what challenges you faced after contracting the illnesses. It's important to note that all interviews took place at the residential places of the study participants, lasting approximately 70–90 min. The interview prompts were developed based on a thorough review of existing literature on tuberculosis, reproductive health, and women's psychosocial well-being, particularly within the South Asian context. Additionally, insights from pilot interviews and consultations with healthcare professionals and social scientists familiar with the cultural context of Kashmir informed the development of questions. The prompts were designed to guide the conversation while allowing participants to introduce new topics relevant to their lived experiences. Furthermore, the interviews were conducted in the local language, Kashmiri, and all of them were audio-recorded.

## Data analysis

In terms of data analysis, we employed qualitative interviews with the participants, which involve debates and deliberations where a researcher carefully and vigilantly motivates and encourages a conversational partner through an extended discussion (23). In the current study, we allowed all participants to freely respond and share as much background information as they felt comfortable. So far as the qualitative interview process is concerned, it has innumerable advantages, and one such advantage is that it comes with the flexibility to make modifications to the questions to meet the knowledge, experience, understanding, and comfort levels of the participants. We transcribed all the interviews verbatim after they concluded. Also, the study implemented a thematic analysis of the data. Thematic analysis was conducted manually without the use of specialized software, following the six-phase framework outlined by Braun and Clarke (2006). This process involved familiarizing with the data through repeated readings of transcribed interviews, generating initial codes by identifying meaningful units of text, and grouping these codes into broader categories to identify themes. The themes were then reviewed for consistency, defined, and named to reflect participants' core experiences. The final analysis explored connections between themes to provide a comprehensive understanding of the participants' lived experiences. To enhance clarity, the main themes identified were: (1) Tuberculosis as an Enduring Experience, including sub-themes such as emotional response to physical suffering, psychological distress, and disruption of familial roles; (2) Tuberculosis and Concerns Around Conceiving, covering infertility-related stigma, disruptions in marital intimacy, and economic repercussions; and (3) Cultural and Societal Influences, highlighting patriarchal norms, fear of disclosure, and limited healthcare access for women in rural areas (21, 22). The present study explored each generated theme to gain a deeper understanding of the participant's experiences as a tuberculosis patient.

## Ethical considerations

To get the endorsement for ethical considerations, the study has received ethical clearance from the “Institutional Ethical Committee” of (anonymized name of institution)*.* The present study adhered to high ethical standards by safeguarding the confidentiality of the study participants' responses and obtaining their consent prior to conducting the research. First and foremost, we obtained approval from each study participant before starting the interview. We thoroughly informed each study participant about the study, its aims, and objectives to obtain their informed consent. Moreover, the researcher informed the participants about the significance of their participation and the nature of their contribution. The researcher respected each study participant's right to privacy and ensured the confidentiality of any private information they requested. The researcher did not interview any of the study participants against their will or force them. We considered the study participants’ confidentiality crucial due to the significant social stigma associated with the topic of study.

## Results

### Outcomes of the research study

Based on the analysis of the data, the researchers drew the results into two sections.

1. Socio-demographic description of the study participants.
2. Two subordinate themes; were (2.1) *Tuberculosis an enduring experience: An Emotional response to physical suffering* (2.2) *Tuberculosis and the concern on conceiving: A case beyond Microbiology*.

#### Socio-demographic representation of the study participants

In the current study, we selected 21 participants. All study participants were married women who had problems conceiving due to genital tuberculosis. Of the 21 study participants, all were extra-pulmonary tuberculosis patients who had tuberculosis either in their genital tract or in the uterus. Regarding the age group of study participants and their inability to conceive due to tuberculosis, the age group of 20's had the highest percentage of participants experiencing difficulties in conception and not conceiving at the time of the interview, accounting for 71.4%, while the age group of 30's accounted for 28.6%. Of the 21 study participants, 33% had studied up to secondary level, 23% had studied up to senior secondary, 19% had studied up to graduation, 4.7% had a master's degree, and 19% could not read or write. Moreover, the study participants' livelihoods and incomes ranged from holding private jobs to running their own home-based businesses. Among the 21 study participants, 47.6% were working in the private sector, 9.5% were government employees, 23.8% were doing home business, and 19% of them were housewives.

The analysis revealed differences based on age and education level. Younger participants, particularly those in their 20s, faced heightened emotional distress due to societal pressure to conceive early, while older participants experienced greater anxiety over delayed conception. Education also played a role, with more educated women showing better awareness of TB treatment and healthcare access. Cross-cutting themes such as economic constraints and healthcare access issues emerged, especially among participants from rural or low-income backgrounds, further intensifying the psychological and social impact of the disease.

### Themes

#### Tuberculosis an enduring experience: An emotional response to physical sufferings

Tuberculosis continues to be a global health problem of grave magnitude, requiring crucial responsiveness (20). Despite extensive efforts to control it, tuberculosis continues to remain intangible, inadequately understood, and emergent, often depicted as an uncontrollable epidemic. All study participants diagnosed with tuberculosis and battling it experienced numerous distressing and disturbing phases after the disease took hold, according to the findings. Upon learning of their illness status, the majority of participants experienced shock and a profound emotional breakdown. Not only did their ill health contribute to the shock and emotional breakdown, but also the significant trauma they experienced from their families (in-laws) and significant others. Because were apprehensive about disclosing the disease due to the trauma they had experienced and the potential loss of their social standing.

Despite having non-communicable tuberculosis, I cover my mouth when my children are around. Even after contracting tuberculosis, I stopped serving them meals. I gave this responsibility to my daughter, who is 16 years old. I don't want to put their lives at risk. Their safety is my first priority, (Participant).

The study participants developed psychosocial dissociation and mental and emotional suffering because of this illness and the stigma it carries with itself. Various discourses have revealed that individuals' understanding and experience of severe illness and ailments deviate from their perception of themselves, their lives, their present, and their future (22, 24). Various studies have indicated that tuberculosis rescinds the taken-for-grantedness of normal living, resulting in distress and anguish. Moreover, numerous authors and specialists in the field posit that sufferings stem from a multitude of interconnected traumas and harms, leading to a loss of individuality, anxiety about one's identity, apprehension about one's future, apprehension about one's self-perception, an apparent absence of coping strategies, a feeling of personal bereavement, and a deficiency of hope (25–29).

Nevertheless, tuberculosis can have devastating psychological and social costs. Patients may experience dejection from their own loved ones and relatives, leading to a broken social life (30). Such discernment and ostracization might result in fretfulness, despair, and a decline in the quality of life, and it is due to these potential consequences that most people tend to hide their illness and suffering (31–33).

‘I had a strong desire to become a mother after my marriage. I started developing severe abdominal aches shortly after my marriage, which, when consulted with a gynecologist, revealed a heavy mass in my uterus. The news of tuberculosis in the uterus dealt me another setback. My husband accompanied me throughout this phase without letting anyone know. When they asked us to restrict any sexual contact until the completion of the treatment, our desire to become parents remained unfulfilled. Despite experiencing emotional distress, my husband provided unwavering support throughout the treatment (Participant).

Research indicates that the diagnosis of tuberculosis elicits significant negative psychological distress, and the process of identifying tuberculosis appears to be a distressing and upsetting event for individuals affected by it (22). Information about a tuberculosis diagnosis often disrupts everyday life, even in the most dire circumstances, triggering a catastrophic event in the lives of the patients (22). In the current study, every participant responded to the tuberculosis diagnosis with a range of emotions, including doubt, apprehension, shudder, grief, desolation, discomfiture, fretfulness, fear of death, concerns for their children's well-being, concerns for the safety and security of loved ones, feelings of vagueness, melancholy, unhappiness, defenselessness, or resentment.

Despite the knowledge that tuberculosis is curable, people's reactions to the disease continue to be shocking. Since then, we cannot deny the fact that most of the lives have been saved, but the psychological, social, and economic impact of this disease has not improved much (24). This study highlights the detrimental effects of tuberculosis on women, primarily due to the numerous roles they play within their families and society. Their illness not only disrupts their lives, but also impacts every role they fulfill, including managing household chores, caring for their family, spouse, and children, and fulfilling other responsibilities both inside and outside the family, such as maintaining friendly relationships with neighbours and relatives, participating in social gatherings and ceremonies, and managing agricultural and work activities, This is why, when women fall ill, it affects not only themselves but also their children, their significant others, and their entire families (34).

#### Tuberculosis and the concern on conceiving: a case beyond microbiology

Tuberculosis is a disease that has a drastic impact on the health of populations. It has a more devious face because it chronically cripples different systems of the body. The reproductive system of females is very vulnerable to this infection, and the disease has been recognised as an important cause of infertility in recent years (35). Infertility is otherwise a common problem, but only couples who have been afflicted with tuberculosis can understand and feel the social stigma, psychological stress, and trauma behind it. As far as the present study is concerned, the researcher comprehended different probes with the study participants who are not able to conceive because of tuberculosis and the repercussions of this disease on their overall well-being. One of the study participants, who had not conceived for 8 years, narrated that

‘I was infertile for around 7 years and was already fighting a battle. I was conceived after 8 years of marriage, and currently, I am in the 6th month of pregnancy. Suddenly, I started developing complications, which was completely unbearable. When consulted with the doctor and through several medical examinations, I was detected with tuberculosis in the uterus (Participant).

Intimacy between the couple is the primary phase of life that a disease like tuberculosis might disturb. Equally, genital tuberculosis and respiratory tuberculosis have adverse and damaging effects on sexual function. It has been found that urogenital tuberculosis is the second most common extra-pulmonary manifestation of tuberculosis, which can cause sexual and reproductive dysfunction (36). Women who have been infected and affected by any type of tuberculosis may face rejection and desertion and are depressed about a vigorous intimate life, a nuptial relationship, and parenthood (37). Considering the following narrative

“I have a 1-and-a-half-year-old son, and I am still in my parents” family after delivery. My first issue was also after several years of marriage and through a lot of efforts from doctors. Soon after delivery, I was infected and diagnosed with tuberculosis. For treatment, I frequently visit the tuberculosis centre along with my younger sister for check-ups and follow-ups. I was very disturbed about whether to talk about it to my husband or not, but somehow he got to know about it, and instead of helping and supporting me, he abandoned me and ordered me to keep our one-and-a-half-year-old son, as he is barely concerned about our health and wellbeing (Participant).

Emotionally, an individual with tuberculosis experienced melancholy, fretfulness, and a compact state of well-being. Though tuberculosis is curative after taking treatment for some time, the person affected may hedge himself or herself from social interactions and connections because of the apprehension of contaminating others. It has also been found that the affected individuals want to avoid the guilt of infecting their beloved partner, which results in affecting sexual enjoyment and sexual freedoms (38). As one of the study participants narrates

‘I left my studies midway because of the extreme domestic care responsibilities. It was very difficult for me to manage my studies. I had a choice-based marriage at the age of 22. After one year of marriage, I had an abortion, and at the same time, I was diagnosed with tuberculosis. Suddenly, my relationship with my husband changed because of the disease. He developed a strong disinclination towards me, and it became a hell-like situation. Everything turned upside down because of the costs of treatment, and I bared myself by starting to work on a pashmina shawl. At one time, I felt like I would commit suicide when I was sent back to my father’s place. Besides, he (the patient’s husband) sent me a divorce notice because I have tuberculosis in the fallopian tubes (Participant).

It was also revealed that the study participants were having complications in conception, which later got examined with tuberculosis. While getting knowledge of tuberculosis, all the study participants were surprised and shocked, which caused imbalances in their normal state of well-being. In the above narratives, it was also revealed that despite the medications and treatment available for tuberculosis, they found it difficult to live a normal life, and after getting treated, they were also unable to regain their families. Thus, tuberculosis is not only about getting diagnosed and treated; it is a long-term illness, and its survivors combat its repercussions later in life. From the above discussion, it was affirmed that the implications of tuberculosis go beyond its medical consequences because, on one hand, the multidimensional understanding and conceptualization of gender helped to understand the specific individual experiences of women, right from the diagnosis of tuberculosis. Moreover, the intersections of various aspects, such as the stigmatization and discernment of tuberculosis, interactions and associations with loved ones, living engagements, motherhood, economic fallouts, marital age, disclosure of tuberculosis status, etc., yield diverse experiences and explanations. To conclude, the case studies stated above display a multiplicity of outcomes, signifying that the experiences of women with tuberculosis related to sexuality and reproduction are intricate and multifaceted.

## Discussion

The socio-cultural fabric of Kashmir, characterized by deep-rooted patriarchal norms, significantly influences the stigma and psychosocial impact of tuberculosis on women. In this context, a woman's ability to conceive often determines her social status and familial acceptance. Women with tuberculosis, especially those experiencing infertility, face not only physical suffering but also social exclusion, emotional distress, and diminished self-worth. These cultural factors uniquely intensify the challenges they face, making it critical to consider the intersection of illness, gender roles, and societal expectations in the region. Tuberculosis is progressively understood as an enduring, prolonged, and protracted illness because of stubborn medical signs of progress in its cure and treatment. The progression from infection to living with this illness for a prolonged time is a long-winded and torturous voyage that suggests a lens to view the sufferings faced by the people affected by this illness through the amalgamation of personal, societal, cultural, and institutional differences, which often add to vulnerabilities to infection as well as to illness (17). Tuberculosis has been seen to affect people of any class, religion, ethnicity, and socioeconomic status; it disproportionately affects the most marginalised and vulnerable sections of society, including women and children (18). Despite having a robust programme for its elimination which serves millions and has saved thousands of lives, we have had numerous successes in the rapid scaling up of diagnosis and treatment (39). But despite the great achievements both in the intervention strategies and in general understanding of the disease, millions of people are still grappling with it from across the globe. The mere following up of the dominant medical paradigm to manage and address tuberculosis for many years has not been good enough to control and manage the disease. Despite being a current and modern crisis, tuberculosis has remained a whispered disease; its survivors are forced to live in silence, denying them the ability to even talk about their insistent and unforgiving condition while it consumes them (40).

Tuberculosis cannot be viewed as a medical problem alone but is considered holistically in the context of socio-economic background (41). Besides, it has also been seen that diseases like tuberculosis, which is prolonged and protracted, ostracise and demote people both from a societal and financial point of view, the repercussions of which are loneliness, seclusion, isolation, and separation both within their own family and outside the world (19, 37). Nonetheless, tuberculosis in women has been found to have a conflicting and inconsistent effect on families and households at large, which results in reducing the economic development of societies (42). Epidemiological evidence no doubt shows the difference in progression of illness between men and women, but women are undoubtedly less reported due to more restrictions on mobility, poverty, lack of knowledge, diminutive social and economic status, and reduced access to health and wellbeing (43–46). In some areas, women are not allowed to travel individually and must be accompanied by someone from the family. This notion also puts women in a situation where, at the same time, they want to go for treatment and also do not want to disclose their illness to anyone (47). As a result, most of them avoid going to health centres due to the fear of exposure. Such prohibitions lead to delays, late diagnosis, and a poorer prognosis for survival (48).

Considering the above themes, the study highlights that diseases like tuberculosis and their long and arduous treatment can lead to major disruptions in physical and psychological health among those affected by them (49). Since the present study was carried out in Kashmir, the popular imagination of tuberculosis has not changed much, and even today, a tuberculosis patient is treated with fear, discrimination, and subjugation. It has been largely found that after tuberculosis status is revealed, a strange silence persists around the patient due to poor understanding of it and also the inability to recognise symptoms and seek effective and timely care without fear and discrimination. Society still keeps archaic and divisive concepts like the patriarchal system and stigma and discrimination against females with tuberculosis, which creates a devastating social and psychological impact (35, 40, 45, 46, 50, 51).

The impact of tuberculosis on the study participants was greater than their inability to conceive. Both of these issues are gruesome for the overall well-being of a woman, yet many of her loved ones provide psychosocial support, but the majority of the study participants in the present study were denied such support. Instead, they were dejected and subjugated, which resulted in broken social lives and social interactions. It has been further found that the presentation and severity of the illness turned out to be more challenging for the study participants who were unable to conceive because of tuberculosis because they were all young and were recently married. They faced a lot of difficulties because, on the one hand, they were ascribed to be mothers, and on the other hand, they got affected by tuberculosis, which continued to influence the circumstances in which they lived during that time. Their plight of misery was extremely distressful because they found it tremendously challenging to disclose (52). While some of them disclosed it to their spouses, the majority concealed their illness status among their families (in-laws) due to the concern of being abandoned (53, 54). It has been found that the impact of tuberculosis on women is often neglected and has been found to be one of the deadliest diseases, taking the lives of women more than other causes of maternal mortality (54).

Moreover, the greatest shortcoming has been the dearth of understanding the patient experience of struggling and enduring tuberculosis. Based on the dominant medical paradigm, it is easily assumed that they have needs and challenges without actually listening to them (55). However, the side effects of over-medicalization and the intensity of stigma that pushes them into silence have also mostly been ignored under this paradigm, which is why they became unable to reintegrate into society even after being cured. It has also been found that after taking drugs for their treatment and after being non-infectious even after two weeks only, the disgrace, humiliation, embarrassment, and indignity persist with it well after it gets cured (56). Moreover, the inability to talk about the disease and the miseries surrounding it causes not only isolation and silence but also an internalisation of the suffering and stigma (57). It has largely been found that tuberculosis patients often experience rejection and social isolation from their loved ones, and that is the attitude that was seen to harm patients much more than the disease itself (51).

Further, the stigma of tuberculosis has been seen taking a deeper toll on the overall quality of life of women, and given our patriarchal society, there have been instances of women being thrown out of their houses, being isolated, neglected, and left to fetch for themselves (24, 58). Moreover, it has also been observed that family members play a very crucial role in helping the patient overcome the disease crisis. The stigma surrounding tuberculosis has had crippling implications not only for the health of the patients affected but also for the overall disease control programmes. It causes extreme psychosocial suffering to the person affected by it, which in turn impacts tuberculosis treatment, completion, and recovery.

## Strengths of the study

1. Tuberculosis is a significant global health issue, and exploring its impact on motherhood contributes to public health understanding and interventions.
2. Understanding how tuberculosis affects motherhood can inform strategies to improve maternal and child health, considering the potential consequences for both.
3. Research on tuberculosis and its impact on motherhood can contribute to epidemiological knowledge, helping identify high-risk populations and guiding preventive measures.

## Limitations of the study

1. Studying the impact of tuberculosis on motherhood may raise ethical concerns, particularly related to conducting research on women who cannot conceive because of the illness.
2. Limited access to comprehensive and reliable data on tuberculosis and its effects on motherhood may hinder the depth and accuracy of the study.
3. Findings may not be universally applicable due to variations in tuberculosis prevalence, healthcare infrastructure, and cultural practices across different regions.

## Research implications

The research on the implications of tuberculosis on conception holds several key implications. It could inform clinical guidelines, enhancing the management of tuberculosis in women of reproductive age with a focus on conceiving, pregnancy, and other related considerations. Additionally, the findings may guide the development of targeted interventions, antenatal care strategies, and family planning programmes to improve maternal health outcomes. The research could serve as a foundation for health education initiatives, influence policy recommendations, and prioritise future research efforts to address the specific challenges faced by women conceiving in the context of tuberculosis. Furthermore, the study may highlight the need for integrating tuberculosis awareness and prevention measures into family planning programmes to address concerns related to conceiving and managing the disease during the reproductive years. Besides this, the identification of gaps in knowledge or areas requiring further investigation may help prioritise future research efforts, directing attention to aspects that are crucial for improving outcomes in this population.

## Conclusion

The present paper tries to reconnoiter the understanding of women affected by tuberculosis, their experiences, and the challenges they face in the course of their illness. It also tries to explore the impact of not being able to conceive on their social relations. The researcher established that the knowledge of tuberculosis brought extreme agony and frustration to all the participants because their normal state of well-being had been disturbed. Tuberculosis, despite affecting some organs of the body, fully damages the patient's psyche because this disease is considered more on the head than on the chest or any part of the body. Because of the complexity involved in tuberculosis and its associated psychological and social implications, the affected persons were subjected to enormous psychological and social distress, regardless of the type of tuberculosis they had been affected by. Knowledge about tuberculosis often causes social ostracism and turbulence in interpersonal relationships, which adversely affects psychosocial well-being.

The study also found that, although people were aware of tuberculosis disease, it is treatable and curable. The previous knowledge that generations are passing along, like stigmatization and isolation, substantially represented the socio-cultural beliefs of society. Moreover, social vulnerability contributed to women's reticence to disclose tuberculosis. Like any other disease that is more fatal than tuberculosis, the disclosure of it is very slight because it carries intense social stigma with it. Although an individual is a social being and needs social support to live, being affected by this illness decreases social care, which is why people hide their diagnosis and feel guilty and ashamed despite knowing that it is treatable and curable.

## Data Availability

The raw data supporting the conclusions of this article will be made available by the authors, without undue reservation.
